# Editorial: New insights in thyroid and Covid-19

**DOI:** 10.3389/fendo.2022.1112695

**Published:** 2022-12-23

**Authors:** Jose Augusto Sgarbi, Celia Regina Nogueira, Gabriela Brenta, Marco Antonio Campinho

**Affiliations:** ^1^ Thyroid Unit, Division of Endocrinology and Metabolism, Department of Medicine, Faculdade de Medicina de Marília, Marilia, Brazil; ^2^ Department of Internal Medicine, Medical School Botucatu, São Paulo State University (UNESP), Botucatu, Brazil; ^3^ Endocrinology Division, Cesar Milstein Hospital, CABA, Buenos Aires, Argentina; ^4^ Faculty of Medicine and Biomedical Sciences, Universidade do Algarve, Faro, Portugal; ^5^ Algarve Biomedical Center-Research Institute, Universidade do Algarve, Faro, Portugal

**Keywords:** COVID-19, thyroid hormone, thyroid disorders, hyperthyroidism, hypothyroidism

The Coronaviruses Disease 2019 (Covid-19) pandemic, caused by severe acute respiratory syndrome coronavirus 2 (SARS-CoV-2), is perhaps the most dramatic threat to human health since the Spanish flu in 1918. Almost 700 million cases and more than 6 million deaths have been reported worldwide by November 20, 2022 (1). The lung is the main affected organ, and the most critical clinical presentation has been characterized by interstitial pneumonia, acute respiratory distress syndrome, multiple organ failure, and death (2).

Multiple endocrine organs, such as the pituitary, pancreas, adrenal, gonads, and thyroid gland, have also been affected (3). Detrimental effects on thyroid function have been reported in patients with and without pre-existing thyroid disease. Nonthyroidal illness syndrome (NTIS), subacute thyroiditis (SAT), Hashimoto’s thyroiditis, and Graves’ disease have been the most frequent thyroid dysfunctions associated with Covid-19 (4). Two major pathophysiological models have been implicated, a direct effect by virus attack causing follicular cells damage and an indirect effect caused by an immune-inflammatory abnormal response to the virus (5). Most recently, thyroid autoimmune diseases have also been reported following Covid-19 vaccination (6).

Thus, thyroid disorders associated with Covid-19 infection or vaccination have emerged as a new focus of research in thyroidology, which has motivated a particular Research Topic by Frontiers Endocrinology. We (the editorial team) invited potential collaborators worldwide from different disciplines to submit their recent research on Covid-19 and thyroid diseases. Eleven articles involving 83 authors were accepted for publication following at least two peer reviews. The published articles fit in Covid-19 and thyroid function- and autoimmunity, analyzing different themes of clinical interest, such as SAT, NTIS, hypothyroidism, pregnancy, vaccination, and autoimmune-inflammatory response.

In a systematic review including 19 studies (17 case reports and 2 case series), Trimboli et al. found that the size and quality of published data are poor and that the clinical presentation of Covid-19-related SAT is like the classic forms, being usually milder and not requiring any specific treatment.

NTIS was addressed in four studies. Zou et al. studied 149 Covid-19 patients and found that NTIS patients (27.5%) had more robust inflammatory responses, such as higher levels of C-reactive protein and erythrocyte sedimentation rate compared to those with non-NTIS. In addition, NTIS was an independent risk factor for Covid-19 severity. Wang et al. showed similar findings in a retrospective study comparing Covid-19 patients with non-Covid-19 patients. They also observed that thyroid dysfunction recovers gradually and spontaneously and tended to be associated with longer viral nucleic acid cleaning time, suggesting a direct effect of the SARS-CoV-2 virus on the gland. Lui et al. included 541 patients without known thyroid disorders with Covid-19; 15.4% had abnormal thyroid function, NTIS being the most frequent. TSH and FT3 levels independently correlated with lymphocyte counts and SARS-Cov-2 viral load. The authors also found that patients who had both lymphopenia and NTIS were more likely to deteriorate than those who had only one alone and those without lymphopenia or NTIS. Finally, in another study including 174 hospitalized patients with Covid-19, Swiatkowska-Stodulska et al. found that FT3 measured at admission was an independent predictor of unfavorable endpoints such as death, mechanical ventilation, vasopressor infusion, and prolonged hospital stay. All these data suggest an essential role of an abnormal immune- and inflammatory response in the pathogenesis of NTIS in Covid-19 patients and its correlation with Covid-19 severity.

Different features of thyroid function were explored in the other three studies. Gerwen et al. did not find any association between pre-existing hypothyroidism with increased risk of hospitalization, mechanical ventilation, or death, indicating that no additional precautions or specific recommendations are needed for patients with hypothyroidism. On the other hand, a retrospective cohort study by Lin et al. showed that pregnant women in their first trimester during the Covid-19 outbreak in Shanghai were at increased risk of having isolated hypothyroxinemia, emphasizing the importance of monitoring thyroid function in pregnant women during and after Covid-19. In addition, Yang and Xu found that postoperative thyroid patients tended to have more mental health problems and less psychological support during the Covid-19 pandemic. Fröhlich and Wahl. presented the readers with a narrative overview of several themes, such as thyroid hormones- and their metabolite’s actions, thyroid dysfunctions in Covid-19, and the potential use of L-T3 and their metabolites in the treatment of severely ill Covid-19 patients.

Finally, two interesting articles addressed the link between Covid-19, thyroid autoimmunity, and vaccination. Lui et al. observed that interferon therapy for Covid-19 was associated with modest increases in thyroid peroxidase (TPO) antibody titers and incidence. Furthermore, incident anti-TPO positivity was more likely to be related to abnormal thyroid function during convalescence, suggesting that clinicians monitor thyroid function and anti-thyroid antibodies among interferon-treated Covid-19 patients. As a complement to the work of Lui et al., an interestingly combined retrospective and prospective study by Huang et al. demonstrated that thyrotropin receptor antibody (TRAB) serum levels of Graves’ disease patients decreased less after inactivated SARS-Cov-2 vaccination and showed an upward trend. Not surprisingly, FT3 and FT4 levels were consistent with it. These data provide evidence for clinicians monitoring TRAB- and thyroid hormone levels after inactivated SARS-Cov-2 vaccination.

Most of the studies in this Research Topic of Frontiers in Endocrinology focus on the thyroid dysfunctions associated with Covid-19, having further advanced the evidence for clinicians in managing adults and pregnant women with- and without pre-existent thyroid dysfunctions. Additionally, new insights into the potential effects of vaccination on thyroid autoimmunity were highlighted.

## Author contributions

JS wrote de manuscript. GB, CN, and MC critically reviewed and approved the final manuscript. All authors contributed to the article and approved the submitted version.
